# The Association Between Elevated Thyroid-Stimulating Hormone Levels and Prolonged Length of Stay Among Adult Diabetic Patients Hospitalized in Internal Medicine Departments: A Large Historical Cohort Study

**DOI:** 10.3390/jcm13226837

**Published:** 2024-11-14

**Authors:** Aviel Kuchar, Tomer Ziv-Baran, Eugene Feigin, Elad Shemesh, Assaf Buch, Roy Eldor, Yona Greenman, Elena Izkhakov

**Affiliations:** 1 Faculty of Medicine, Tel Aviv University, Tel Aviv 6997801, Israel; avielk1@gmail.com (A.K.); eugenef@tlvmc.gov.il (E.F.); roye@tlvmc.gov.il (R.E.); yonagr@tlvmc.gov.il (Y.G.); 2 Department of Epidemiology and Preventive Medicine, School of Public Health, Faculty of Medicine, Tel Aviv University, Tel Aviv 6997801, Israel; zivtome@tauex.tau.ac.il; 3 Institute of Endocrinology, Diabetes, Metabolism and Hypertension, Tel Aviv Sourasky Medical Center, 6 Weizmann Street, Tel Aviv 6423906, Israel; eladshe@tlvmc.gov.il (E.S.); buchasaf@gmail.com (A.B.); 4 Department of Nutritional Sciences, School of Health Sciences, Ariel University, Ariel 40700, Israel

**Keywords:** type 2 diabetes mellitus, thyroid, TSH, hypothyroidism, hospitalization, LOS

## Abstract

**Background/Objectives**: Type 2 diabetes mellitus (DM2) and hypothyroidism are two of the most common endocrine disorders in clinical practice. Hospital length of stay (LOS) is a quality metric of the health systems. We evaluated the association between elevated thyroid-stimulating hormone (TSH) levels and prolonged LOS among all adult patients (age ≥ 18 years) with DM admitted to our Internal Medicine departments between 2014 and 2022. **Methods**: Data on patient characteristics, LOS, and in-hospital mortality were collected. A TSH level > 4.7 μIU/mL was considered as being elevated. A LOS > 75th percentile and in-hospital mortality were defined as being prolonged. Univariate and multivariable analyses were applied, and propensity score matching controlled for differences between patients with normal and those with elevated TSH levels. **Results**: Of the 19,066 study participants (median age 75.6 years, IQR 75.9–83.3), 1524 (7.9%) had elevated TSH levels, and prolonged LOS was significantly more common among them (before matching: 38.6% vs. 29.1%, *p* < 0.001; after matching: 38.7% vs. 32.6%, *p* = 0.001). After adjustment for potential confounders, elevated TSH levels were also associated with prolonged LOS (OR = 1.22, 95% CI 1.07–1.39, *p* = 0.002). **Conclusions:** Elevated TSH levels in diabetic patients hospitalized in Internal Medicine departments are associated with prolonged LOS, emphasizing the importance of identifying hypothyroidism among them.

## 1. Introduction

Hypothyroidism and type 2 diabetes mellitus (DM2) are prevalent endocrine disorders among hospitalized patients, with approximately 9.0–11.1% diagnosed as having DM2 and 1–2% as having primary hypothyroidism [1,2]. DM2 is associated with adverse hospitalization outcomes, including prolonged length of stay (LOS) and higher rates of in-hospital mortality [3,4,5]. Elevated thyroid-stimulating hormone (TSH) levels, a hallmark of hypothyroidism, are also associated with adverse outcomes and LOS in diverse patient populations, including the elderly and those with heart failure, kidney disease, and hyponatremia [6,7,8,9,10,11,12,13]. The importance of the concurrent presence of these morbidities lies in the high prevalence of hypothyroidism among individuals with DM2, with up to 14.7% of them having at least mild thyroid dysfunction, most commonly hypothyroidism [11,14]. Older age, obesity, and the presence of thyroid autoantibodies have been identified as risk factors for the development of hypothyroidism among DM2 patients [14,15]. The coexistence of thyroid dysfunction and DM2 has been reported in several studies that have identified an association between low levels of thyroid hormones and diabetes imbalance, insulin resistance, and the development of diabetes [16,17,18].

Hospital LOS is a quality metric in health systems. Prolonged LOS may be associated with increased inpatient complications, some of which could be prevented. It can also worsen the experience for both patients and staff. Ward strain occurs when demand exceeds capacity, and it is associated with poorer patient outcomes. Reducing LOS improves bed availability, allowing hospitals to better manage admissions, both planned and unplanned, as well as transfers between departments. However, shorter stays may be associated with post-discharge adverse outcomes. As a result, hospitals are constantly seeking ways to balance between delivering the best possible care, ensuring safe discharges, and avoiding unnecessary extended stays [19].

While the association between elevated TSH levels and adverse patient outcomes is well established in some patient populations, the association between elevated TSH levels and prolonged hospital LOS has not yet been studied among patients with DM2 [20,21,22,23,24,25,26,27,28]. Therefore, this study aimed to determine whether an elevated TSH level is associated with prolonged hospital LOS in a large number of individuals with DM2 hospitalized in Internal Medicine departments.

## 2. Methods

### 2.1. Study Design and Population

This historical cohort study was performed at a tertiary university-affiliated 1500-bed medical center. Included were all patients with DM2 (aged ≥ 18 years) admitted to the medical center between 2014 and 2022 and hospitalized for any reason in Internal Medicine departments. Patients with TSH levels below the normal range (<0.4 mU/L) and those without a TSH measurement were excluded. The local institutional review board approved the study (0691-22-TLV) and waived informed consent for this anonymized retrospective analysis.

### 2.2. Data Source, Measurements, and Variables

Data were obtained by means of MDClone, a powerful query system that utilizes data from patient records and enables the construction of large patient cohorts based upon demographic, diagnostic, and therapeutic events [29]. Extracted data included age, sex, body mass index (BMI), cardiovascular comorbidities (hypertension, ischemic heart disease, congestive heart failure, peripheral vascular disease, and previous stroke), chronic obstructive pulmonary disease, liver disease, chronic kidney disease, malignancy, a documented diagnosis of hyperthyroidism or treatment with levothyroxine, the Charlson comorbidity index (CCI) [30], Norton score [31], and results from the first blood test upon hospital arrival. Extracted data also included the main diagnosis at admission divided into seven categories (infectious, cardiovascular, neurological, gastrointestinal, malignancy-related, blood test abnormalities, and others).

Blood test results included complete blood count, C-reactive protein (CRP), glucose, and albumin. Hemoglobin A1c (HbA1c) was included if the test was performed within the first week of hospitalization. The 2021 CKD-EPI equation was used to calculate the estimated glomerular filtration rate (eGFR) from the first creatinine measurement [32]. A prolonged LOS was defined as the upper quartile of the LOS. Patients who died during the initial days of hospitalization (less than the upper quartile) were considered as having a prolonged LOS in order to prevent selection bias of severely ill patients, which likely would have had extended stays had they survived.

### 2.3. Laboratory Methods

Blood samples were collected in tubes by experienced nurses using standard venipuncture techniques. The tubes were then transferred to the medical center’s central laboratory. Blood cells counts were performed by the Beckman Coulter UniCel. The blood chemistry tests employed serum separator collection vacuum plastic tubes with a gel separator. The samples were centrifuged for 10 min at 2000× *g*. Chemistry blood tests were analyzed by the automated clinical chemistry analyzers ADVIA system, and TSH blood tests (normal range 0.4–4.7 mU/L) were analyzed by the ADVIA Centaur Immunoassay System (Siemens Healthcare Diagnostics Inc., Tarrytown, NY, USA). All tests were evaluated by means of standard methods according to the manufacturers’ recommendations. An elevated TSH level was defined as a TSH level higher than 4.7 mU/L.

### 2.4. Statistical Analysis

Categorical data were summarized as frequencies and percentages. The distribution of the continuous variables was evaluated by histograms and Q-Q plots, and they were reported as medians and interquartile ranges (IQRs). A multivariable analysis as well as matching techniques were applied, since the characteristics of the patient populations with normal and elevated TSH levels were assumed to be different. The standardized mean difference (SMD) was used to compare patients with and those without elevated TSH levels, before and after matching. A standardized difference of <0.1 was taken as being negligible, and one between 0.1 and 0.2 as being small. The normal and elevated TSH groups were matched according to the probability of having elevated TSH levels. We employed a logistic regression model to calculate the probability (propensity score) according to the following parameters: sex, hypertension, dyslipidemia, ischemic heart disease (IHD), congestive heart failure (CHF), previous stroke, peripheral vascular disease (PVD), chronic kidney disease (CKD), chronic obstructive pulmonary disease (COPD), history of malignancy, liver disease, hypothyroidism or treatment with levothyroxine, admission diagnosis, age, BMI, hemoglobulin (Hb), white blood count (WBC), neutrophil-to-lymphocyte ratio (NLR), platelets (PLTs), glucose, eGFR, albumin, CRP, the Charlson score, and Norton score. Fuzzy matching without replacement was also conducted. An absolute difference in the propensity score of up to 5% (on a scale of 0 to 100% and using a matching tolerance/caliper) was taken as being acceptable for matching.

Before matching, the association between elevated TSH levels and prolonged LOS was evaluated by a multivariable logistic regression while controlling for possible confounders. The model contained three blocks, where elevated TSH was forced into the first block and age and sex were forced into the second block. Dyslipidemia, IHD, CHF, previous stroke, PVD, CKD, COPD, history of malignancy, liver disease, hypothyroidism or treatment with levothyroxine, admission diagnosis, age, BMI, Hb, WBC, NLR, PLTs, glucose, eGFR, albumin, CRP, the CCI, and Norton score were considered as potential variables for inclusion in the model using the forward method (the Wald test was used and *p* < 0.05 was the criterion for inclusion) and forced into the third block. After matching, prolonged LOS findings were compared between the normal and elevated TSH groups by means of the McNamar test. All statistical tests were two-sided, and *p* < 0.05 was considered statistically significant. Statistical analyses were performed using SPSS software (IBM SPSS Statistics for Windows, version 29, IBM Corp., Armonk, NY, USA, 2022).

## 3. Results

### 3.1. Patient Characteristics

A total of 19,066 hospitalized patients with DM2 were included in the final analysis. Of them, 1524 (7.9%) had elevated TSH levels (>4.7 mU/L). The baseline characteristics of patients with TSH levels within the normal range and those with elevated TSH levels before and after matching are summarized in Table 1. Before matching, there were significant differences (SMD > 0.1) between the normal and elevated TSH groups. The latter had a lower proportion of males (42.8% vs. 56.1%), the patients were older (median age 77.3 vs. 75.6 years), and that group had higher proportions of CHF (26.4% vs. 18.0%) and CKD (18.2% vs. 13.6%). In addition, the elevated TSH group had higher platelet counts and lower Hb, glucose, and albumin levels. A total of 603 of the 2217 levothyroxine-treated patients (27.2%) had elevated TSH levels. The overall median LOS was 4.17 days (IQR 2.33–8.33), and the in-hospital mortality rate was 7.2% (6.9% in the normal TSH group and 10.7% in the elevated TSH group).

### 3.2. Association Between Elevated TSH Levels and Prolonged LOS—Unmatched Groups

**Figure 1:**
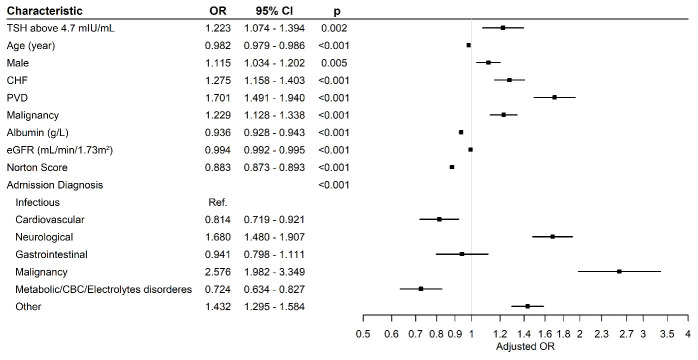
Multivariable analyses of predictors for prolonged length of hospital stay. CBC = complete blood count; CHF = congestive heart failure; CI = confidence interval; eGFR = estimated glomerular filtration rate; OR = odds ratio; PVD = peripheral vascular disease; TSH = thyroid-stimulating hormone.

Before matching, 5112 (29.1%) of the patients in the normal TSH group had prolonged LOS, while the proportion in the elevated TSH group was significantly higher (*n* = 589, 38.6%, *p* < 0.001). After adjustment for potential confounders, elevated TSH levels were also associated with a prolonged LOS (odds ratio [OR] 1.22, 95% confidence interval [CI] 1.07–1.39, *p* = 0.002). The regression model is presented in Figure 1. Previous levothyroxine treatment did not affect the LOS (OR 0.93, 95% CI 0.72–1.21, *p* = 0.611).

### 3.3. Association Between Elevated TSH Levels and Prolonged LOS—Matched Groups

**Figure 2:**
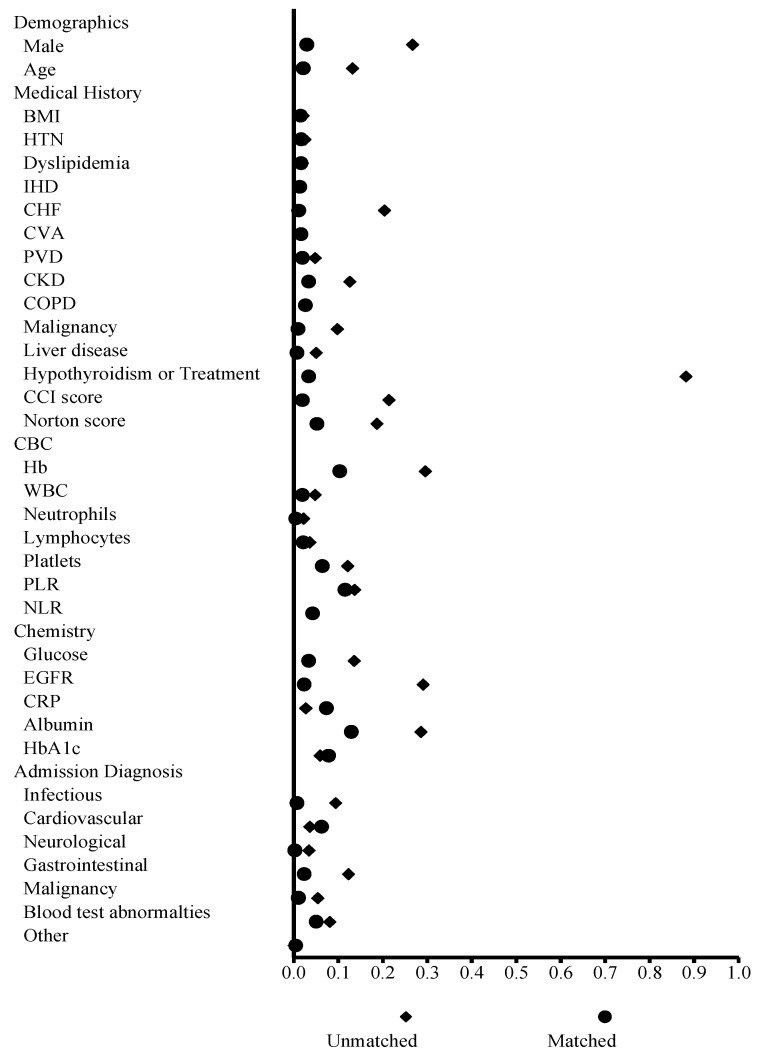
Standardized mean difference in groups before and after matching. BMI = body mass index; CCI = Charlson comorbidity index; COPD = chronic obstructive pulmonary disease; CVA = cerebrovascular accident; CHF = congestive heart failure; CKD = chronic kidney disease; CRP = C-reactive protein; eGFR = estimated glomerular filtration rate; Hb = hemoglobin; HTN = hypertension; IHD = ischemic heart disease; NLR = neutrophil-to-lymphocyte ratio; PLR = platelet-to-lymphocyte ratio; PVD = peripheral vascular disease.

The groups were matched in order to address differences in baseline characteristics, resulting in 1266 patients in each group. The groups were comparable across most characteristics (SMD < 0.1), with the exception of Hb (SMD = 0.103), serum albumin (SMD = 0.129), and the platelet-to-lymphocyte ratio (SMD = 0.115, Table 1 and Figure 2). After matching, 413 (32.6%) patients in the normal TSH group and 490 patients (38.7%) in the elevated TSH group had a prolonged LOS (*p* = 0.001).

### 3.4. Association Between Mildly Elevated TSH Levels, Moderately and Highly Elevated TSH Levels, and Prolonged LOS—Subanalysis

Overall, 1196 patients had mildly elevated TSH levels (>4.7–10 mU/L) and 328 had moderately and highly elevated TSH levels (>10 mU/L). The highest rate of prolonged LOS was measured in patients with moderately and highly elevated TSH levels (45.4%), followed by patients with mildly elevated TSH levels (36.8%) and those with normal TSH levels (29.1%, *p* < 0.001). Similar results were obtained in the multivariate analysis (moderately + highly elevated vs. normal: OR 1.43, 95% CI 1.09–1.86, *p* = 0.034; mildly elevated vs. normal: OR 1.17, 95% CI 1.01–1.36, *p* = 0.009).

## 4. Discussion

The study included 19,066 patients with DM2 and a sub-cohort of 2532 matched non-DM patients. The results showed a significant association between an elevated TSH level and a prolonged LOS (OR 1.22, 95% CI 1.07–1.39, *p* = 0.002). This finding aligns with previous research that demonstrated an association between elevated TSH levels and various other adverse outcomes [6,7,8,9,10,19,20,21,22,23,24,25,26,27]. To the best of our knowledge, this is the first study to explore this association within a large cohort of patients with DM2 hospitalized in Internal Medicine departments. The overall median LOS was 4.17 days. Previous studies that examined the LOS among patients with DM2 showed a similar median LOS of 4.8–6 days [4,33].

Male sex (OR = 1.115, *p* = 0.005), CHF (OR = 1.275, *p* < 0.001), PVD (OR = 1.701, *p* < 0.001), and malignancy (OR = 1.229, *p* < 0.001) were also independent predictors for increased LOS in our current study. In a large cohort study by Kahlid et al., male sex (HR = 1.11, 95% CI = 1.09–1.13), cardiovascular complications (HR = 1.87, 95% CI = 1.81–1.93), peripheral circulatory disorders (HR = 1.12, 95% CI = 1.09–1.16), and cancer (HR = 1.61, 95% CI = 1.57–1.66) were associated with an elevated risk of hospitalization and mortality [33]. In a prediction model for adverse outcomes in hospitalized patients with DM2 developed by Nirantharakumar et al., decreased albumin levels (albumin 25–34 g/L, OR = 1.74, albumin < 25 g/L OR = 2.64, *p* < 0.001) and reduced eGFR (eGFR < 30 mL/min/1.73 m^2^, OR = 1.31, *p* < 0.001) were associated with extended LOS and mortality, supporting our current findings (albumin OR = 0.936, eGFR OR = 0.994, *p* < 0.001) [34]. However, those studies did not consider the TSH levels.

A possible explanation for the association between elevated TSH and prolonged LOS could be a complex interplay between endocrine systems. In addition, the association can be at least partially explained by the fact that TSH receptors were found not only in the pituitary, the hypothalamus, and the thyroid cells but also in the skin, the kidneys, the cardiac and skeletal muscles, the periorbital tissue, and the immune system, as well as in bone [35,36]. The potential mechanism and impact of elevated TSH on the different sites of TSH receptor expression remain controversial [35,36].

It is well established that DM2 and thyroid dysfunction individually contribute to a range of adverse health outcomes, including cardiovascular complications, metabolic disturbances, and impaired immune function [3,4,5,6,7,8,9,10,37]. The coexistence of diabetes and hypothyroidism may lead to a synergistic effect, exacerbating the underlying pathophysiological processes and increasing the risk of adverse outcomes. For example, the influence of hypothyroidism on cardiovascular health coupled with the increased cardiovascular risk in DM2 may contribute to many unfavorable outcomes, as well as to prolonged hospitalization [38,39]. These factors collectively highlight the intricate relationship between diabetes and hypothyroidism, underscoring the need for further research to elucidate the specific mechanisms driving the prolonged hospital stays in this patient population.

This study has several limitations. First, its retrospective design introduces potential biases and confounders. For example, potential confounders, such as DM2 medication prior to hospitalization, were not coded, and therefore could not be included. However, other relevant confounders, such as co-morbidities, admission diagnosis, the Norton score, and the CCI were available and included in the current study. Second, having been conducted at a single center may restrict the generalizability of the findings to other medical centers, which may have different treatment protocols. However, the median LOS of this study was comparable to that reported in previous studies. Third, our study relies upon data extracted from the medical center’s data warehouse rather than a direct assessment of patient files. That said, the data stored in the warehouse are directly acquired from the patient’s electronic medical records, which are used for the patients’ routine follow-up and treatment. Moreover, data from similar sources have been used in previous studies. Fourth, a potential limitation lies in a selection bias of the study participants having been limited to patients that had undergone TSH testing upon hospitalization. Patients who were not similarly tested may have exhibited different clinical characteristics compared to those who were. However, TSH testing together with the other blood results reported in this study are included in the basic hospitalization panel. Therefore, the number of diabetic patients hospitalized without TSH testing was probably minimal. Finally, the absence of thyroid peripheral hormone-free T4 levels, which are not included in the basic hospitalization panel, does not allow for distinguishing between subclinical and overt hypothyroidism.

A key strength of this study lies in its incorporation of a large cohort of hospitalized patients spanning an 8-year period, thus ensuring robust statistical power. Another strength lies in its comprehensive consideration of various potential confounders, such as well-established patient co-morbidity scores (the Norton scale and the CCI), and that it also included the admission diagnosis and blood test results [30,40]. Finally, a propensity score was used for matching, and a large number of matched pairs were compared to further validate the study results.

In conclusion, our analysis of a large cohort of hospitalized patients with DM2 revealed that an elevated TSH level was associated with a prolonged LOS. This finding emphasizes the significance of considering the coexistence of hypothyroidism in patients with DM2 in the context of patient outcomes in the hospital setting. We therefore suggest the routine inclusion of TSH as part of the hospital admission laboratory panel of patients with DM hospitalized in Internal Medicine departments. Future studies are needed to identify whether thyroid hormone replacement therapy will lead to a shorter LOS in hospitalized DM2 patients with elevated TSH levels.

## Figures and Tables

**Table 1:** Baseline characteristics of patients with TSH levels within the normal range and elevated TSH levels before and after matching.

	Unmatched			Matched		
Variable	Normal TSH	Elevated TSH	SMD	Normal TSH	Elevated TSH	SMD
Number of patients	17,542	1524		1266	1266	
TSH (mU/L) median (IQR)	1.58 (1.0–2.4)	6.52 (5.4–9.1)		1.75 (1.1–2.7)	6.49 (5.4–9.1)	
Demographics						
Male, *n* (%)	9838 (56.1%)	653 (42.8%)	0.267	573 (45.3%)	555 (43.8%)	0.029
Age (years), median (IQR)	75.6 (66.9–83.4)	77.3 (66.7–84.9)	−0.132	77.8 (69.6–84.3)	76.9 (68.6–84.3)	0.021
Medical history						
BMI, median (IQR)	27.0 (24.1–30.7)	26.9 (23.6–30.9)	0.021	27.0 (24.1–30.5)	27.0 (23.7–31.0)	0.015
HTN, *n* (%)	12,707 (72.4%)	1087 (71.3%)	0.025	915 (72.3%)	906 (71.6%)	0.016
Dyslipidemia, *n* (%)	10,031 (57.2%)	857 (56.2%)	0.019	731 (57.7%)	721 (57.0%)	0.016
IHD, *n* (%)	5674 (32.3%)	501 (32.9%)	−0.011	431 (34.0%)	423 (33.4%)	0.013
CHF, *n* (%)	3149 (18.0%)	402 (26.4%)	−0.204	316 (25.0%)	322 (25.4%)	−0.011
CVA, *n* (%)	2371 (13.5%)	198 (13.0%)	0.016	161 (12.7%)	168 (13.3%)	−0.016
PVD, *n* (%)	1560 (8.9%)	157 (10.3%)	−0.048	119 (9.4%)	126 (10.0%)	−0.019
CKD, *n* (%)	2381 (13.6%)	277 (18.2%)	−0.126	214 (16.9%)	230 (18.2%)	−0.033
COPD, *n* (%)	2178 (12.4%)	202 (13.3%)	−0.025	155 (12.2%)	166 (13.1%)	−0.026
Malignancy, *n* (%)	4297 (24.5%)	439 (28.8%)	−0.098	359 (28.4%)	364 (28.8%)	−0.009
Liver disease, *n* (%)	880 (5.0%)	94 (6.2%)	−0.050	80 (6.3%)	78 (6.2%)	0.007
Hypothyroidism or treatment, *n* (%)	1866 (10.6%)	720 (47.2%)	−0.882	578 (45.7%)	599 (47.3%)	−0.033
CCI score, median (IQR)	5 (4–7)	6 (4–7)	−0.214	5 (4–7)	6 (4–7)	−0.019
Norton score, median (IQR)	18 (14–20)	17 (13–19)	0.187	17 (14–19)	17 (14–19)	0.052
Laboratory values, median (IQR)						
Hb, g/dL	12.0 (10.5–13.4)	11.3 (9.7–12.7)	0.296	11.6 (10.0–12.9)	11.2 (9.8–12.7)	0.103
WBC, 10^3^/μL	8.5 (6.6–11.3)	8.5 (6.4–11.2)	−0.048	8.5 (6.7–11.3)	8.4 (6.3–11.2)	−0.019
Neutrophils, 10^3^/μL	5.9 (4.3–8.6)	5.9 (4.2–8.5)	−0.022	6.0 (4.3–8.7)	5.8 (4.1–8.5)	0.004
Lymphocytes, 10^3^/μL	1.4 (0.9–1.9)	1.3 (0.9–2.0)	−0.036	1.3 (0.9–1.9)	1.3 (0.9–2.0)	−0.021
Platlets, 10^3^/μL	214 (166–275)	224 (170–290)	−0.121	217 (166–285)	226 (171–291)	−0.064
PLR	154 (106–238)	161 (110–267)	−0.137	162 (108–249.3)	162 (110–267)	−0.115
NLR	4.3 (2.6–7.8)	4.4 (2.5–8.0)	−0.043	4.4 (2.6–7.9)	4.3 (2.5–8.0)	−0.042
Glucose (mg/dL)	137 (108–187)	128 (100–174)	0.136	131 (104–178)	128 (100–174)	0.033
eGFR (mL/min/1.73 m^2^)	73.2 (49.2–92.1)	61.8 (39.5–85.6)	0.291	63 (42.0–89.0)	63 (41.0–86.0)	0.023
CRP wide range (mg/L)	25.3 (6.4–101.0)	30.6 (8.8–103.1)	−0.027	24.5 (6.9–89.3)	29.4 (8.5–103.4)	−0.073
Albumin (g/L)	38.0 (34.3–41.0)	37.0 (32.3–40.0)	0.286	37 (34.0–40.0)	37 (33.0–40.0)	0.129
HbA1c (%)	7.0 (6.3–8.2)	6.9 (6.2–8.1)	0.059	6.9 (6.2–7.9)	6.9 (6.2–8.1)	−0.078
Admission diagnosis						
Infectious, *n* (%)	5295 (30.2%)	396 (26.0%)	0.094	335 (26.5%)	331 (26.1%)	0.007
Cardiovascular, *n* (%)	3126 (17.8%)	251 (16.5%)	0.036	239 (18.9%)	209 (16.5%)	0.062
Neurological, *n* (%)	2064 (11.8%)	163 (10.7%)	0.034	143 (11.3%)	144 (11.4%)	−0.002
Gastrointestinal, *n* (%)	993 (5.7%)	135 (8.9%)	−0.123	104 (8.2%)	112 (8.8%)	−0.023
Malignancy, *n* (%)	273 (1.6%)	35 (2.3%)	−0.054	29 (2.3%)	31 (2.4%)	−0.010
Blood test abnormalities, *n* (%)	1883 (10.7%)	204 (13.4%)	−0.081	151 (11.9%)	172 (13.6%)	−0.050
Other, *n* (%)	3908 (22.3%)	340 (22.3%)	−0.001	265 (20.9%)	267 (21.1%)	−0.004

BMI = body mass index; CCI = Charlson comorbidity index; COPD = chronic obstructive pulmonary disease; CVA = cerebrovascular accident; CHF = congestive heart failure; CKD = chronic kidney disease; CRP = C-reactive protein; eGFR = estimated glomerular filtration rate; Hb = hemoglobin; HTN = hypertension; IHD = ischemic heart disease; NLR = neutrophil-to-lymphocyte ratio; PLR = platelet-to-lymphocyte ratio; PVD = peripheral vascular disease; SMD = standard mean difference.

## Data Availability

Data are available from the corresponding author upon reasonable request and according to approval of the local ethics committee.

## Abbreviations

BMI	Body mass index
CCI	Charlson comorbidity index
COPD	Chronic obstructive pulmonary disease
CVA	Cerebrovascular accident
CHF	Congestive heart failure
CKD	Chronic kidney disease
CRP	C-reactive protein
DM2	Type 2 diabetes mellitus
eGFR	Estimated glomerular filtration rate
Hb	Hemoglobin
HTN	Hypertension
IHD	Ischemic heart disease
IQR	Interquartile range
LOS	Length of stay
NLR	Neutrophil-to-lymphocyte ratio
PLR	Platelet-to-lymphocyte ratio
PLT	Platelets
PVD	Peripheral vascular disease
SMD	Standard mean difference
TSH	Thyroid-stimulating hormone
WBC	White blood count
